# Examining arterial pulsation to identify and risk-stratify heart failure subjects with deep neural network

**DOI:** 10.1007/s13246-023-01378-6

**Published:** 2024-02-15

**Authors:** Chieh-Chun Huang, Shih-Hsien Sung, Wei-Ting Wang, Yin-Yuan Su, Chi-Jung Huang, Tzu-Yu Chu, Shao-Yuan Chuang, Chern-En Chiang, Chen-Huan Chen, Chen-Ching Lin, Hao-Min Cheng

**Affiliations:** 1 Institute of Biomedical Informatics, National Yang Ming Chiao Tung University College of Medicine, Taipei, Taiwan; 2 Division of Cardiology, Department of Internal Medicine, Taipei Veterans General Hospital, 112, No. 201, Sec. 2, Shih-Pai Road, Taipei, Taiwan; 3 School of Medicine, National Yang Ming Chiao Tung University College of Medicine, Taipei, Taiwan; 4 Center for Evidence-Based Medicine, Taipei Veterans General Hospital, Taipei, Taiwan; 5 Institute of Population Health Science, National Health Research Institute, Miaoli, Taiwan; 6 General Clinical Research Center, Taipei Veterans General Hospital, Taipei, Taiwan; 7 National Yang Ming Chiao Tung University College of Medicine, Taipei, Taiwan; 8 Institute of Public Health, National Yang Ming Chiao Tung University College of Medicine, Taipei, Taiwan; 9 Department of Medical Education, Taipei Veterans General Hospital, Taipei, Taiwan

**Keywords:** Heart failure, Deep learning, Pulse wave analysis, Pressure waveform

## Abstract

Hemodynamic parameters derived from pulse wave analysis have been shown to predict long-term outcomes in patients with heart failure (HF). Here we aimed to develop a deep-learning based algorithm that incorporates pressure waveforms for the identification and risk stratification of patients with HF. The first study, with a case–control study design to address data imbalance issue, included 431 subjects with HF exhibiting typical symptoms and 1545 control participants with no history of HF (non-HF). Carotid pressure waveforms were obtained from all the participants using applanation tonometry. The HF score, representing the probability of HF, was derived from a one-dimensional deep neural network (DNN) model trained with characteristics of the normalized carotid pressure waveform. In the second study of HF patients, we constructed a Cox regression model with 83 candidate clinical variables along with the HF score to predict the risk of all-cause mortality along with rehospitalization. To identify subjects using the HF score, the sensitivity, specificity, accuracy, F1 score, and area under receiver operating characteristic curve were 0.867, 0.851, 0.874, 0.878, and 0.93, respectively, from the hold-out cross-validation of the DNN, which was better than other machine learning models, including logistic regression, support vector machine, and random forest. With a median follow-up of 5.8 years, the multivariable Cox model using the HF score and other clinical variables outperformed the other HF risk prediction models with concordance index of 0.71, in which only the HF score and five clinical variables were independent significant predictors (p < 0.05), including age, history of percutaneous coronary intervention, concentration of sodium in the emergency room, *N*-terminal pro-brain natriuretic peptide, and hemoglobin. Our study demonstrated the diagnostic and prognostic utility of arterial waveforms in subjects with HF using a DNN model. Pulse wave contains valuable information that can benefit the clinical care of patients with HF.

**Supplementary Information:**

The online version contains supplementary material available at 10.1007/s13246-023-01378-6.

## Introduction

Heart failure (HF) is a major public health problem, with a high prevalence of approximately 2% in the adult population worldwide [1]. The risk of morbidity and mortality among patients with HF remains high despite advanced medical treatment and prevention [2, 3]. Accurate diagnosis and prognostic assessment play central roles in determining high-risk groups in patients with HF by determining preventable factors and designing corresponding strategies for its management given that treatment strategies targeting risk factors, including hypertension, diabetes, and obesity, appear to effectively alleviate its progression [4, 5].

Pulse wave analysis (PWA) is a technique that involves waveform parameters such as augmentation index and central pressure [6, 7]. Applanation tonometry is a non-invasive technique that can be used to obtain continuous arterial pressure waveforms (PWs) [8, 9] whose magnitude and features are influenced by left ventricular (LV) performance, afterload, and arterial stiffness, thereby reflecting the physiological status of the arterial system [10]. Based on the theory that parameters derived from PWA are associated with alterations in vessel wall properties with aging or disease [11–14], PWA was used in previous studies to assess HF [15–20]. However, its utility with modern artificial intelligence (AI) technology for patients with HF has not been investigated.

Deep learning, a machine learning method that performs classification from a large dataset using nonlinear transformation, is commonly used for supervised learning in many fields [21]. With the growing technology of AI [22, 23], the current study aimed to develop a new deep learning model to improve the accuracy of diagnostic and prognostic predictions for patients with HF using AI algorithms and conventional tabular parameters obtained from clinical data. In this study, we constructed a deep neural network (DNN) model that utilizes hidden information within the PWs to identify patients with HF and even to stratify their mortality risks.

**Fig. 1:**
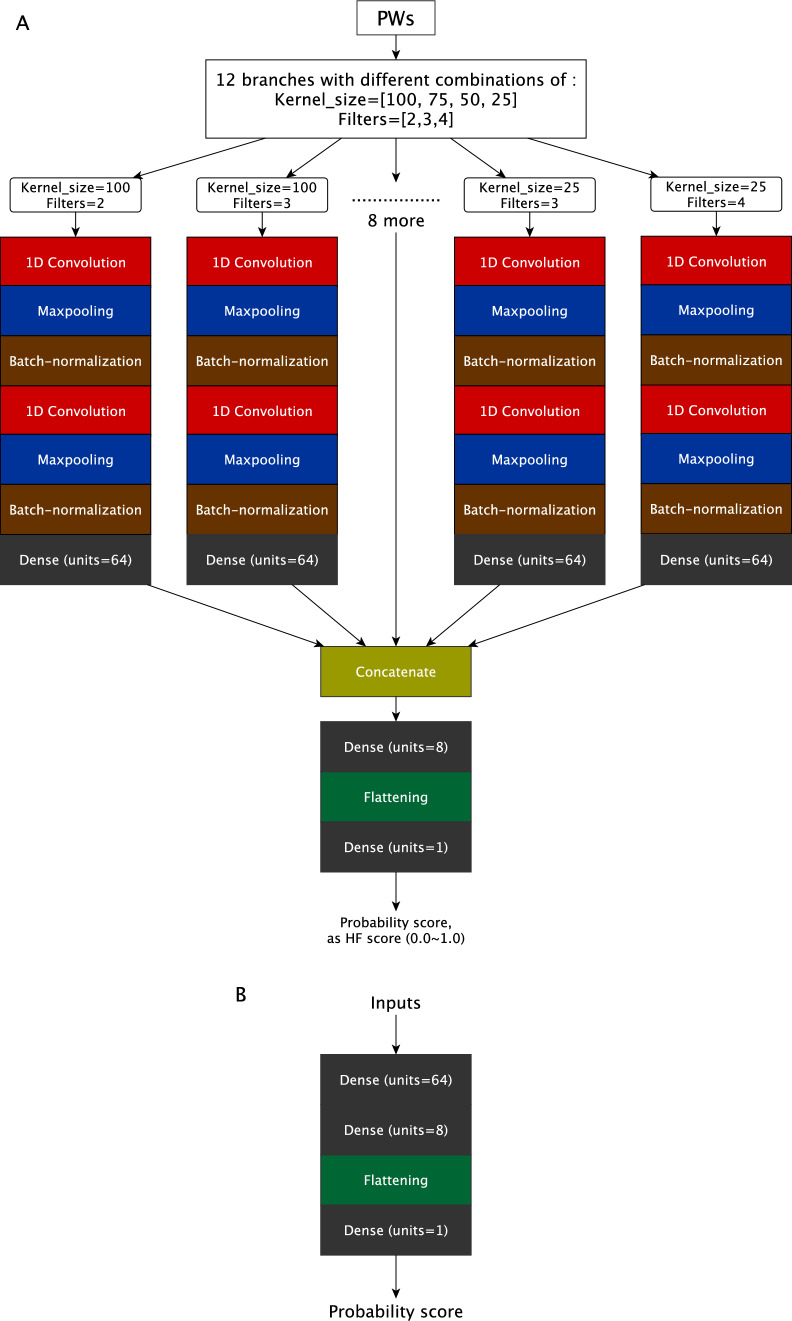
Structure of our DNN model. **A** The Conv1D structure for PWs in 12 branches with different combinations of kernel and filter size, which are respectively [100, 50, 75, 125] and [2–4]. **B** FCL structure for comparison

The functioning of the Deep Neural Network (DNN) is derived from the Conv1D architecture, which is influenced by the transmission of signals in biological neurons. Neural networks, which aim to replicate the functional principles of the brain, consist of algorithmic components such as convolution layers, pooling layers, and fully-connected layers. These components are visually represented in Fig. 1 and Supplementary Fig. 3. The primary aim of the network is to establish a mapping between inputs, which are factors or predictors, and outputs, which are results or outcomes, by identifying and understanding their interrelationships. To gain a more comprehensive understanding of Convolutional Neural Networks (CNNs), here we introduce another article of how CNN works with scientific clue and the development with assistances from other theories [24].

## Methodology

### Data collection from previous research

The current data consisted of two datasets, both of which were approved by the Institutional Review Boards of Taipei Veterans General Hospital and National Health Research Institutes, Taiwan under the numbers 2022-08-006CC and EC1060513; baseline characteristics are shown in Table 1. The present investigation employed a case–control design, comprising an initial cohort of 431 patients who were diagnosed with heart failure and exhibited typical symptoms (referred to as the first dataset). Additionally, a distinct group of 1545 control participants without a previous heart failure diagnosis were included (referred to as the second dataset). It is imperative to acknowledge the potential bias that may emerge due to this discrepancy in the distribution of data [25].

**Table 1:** Baseline characteristics of HF and non-HF patients

Characteristics	HF patients (N = 431)	non-HF control subjects (N = 1545)	p-value
Sex			
Male	333 (77.3%)	728 (47.1%)	﻿ < 0.001
Female	98 (22.8%)	817 (52.9%)	
Age, years	70.0 ± 17.2	60.6 ± 12.3	< 0.001
Age, groups			
< 50	74	299	0.306
50 ~ 59	58	427	< 0.001
60 ~ 69	65	469	< 0.001
70 ~ 79	86	260	0.131
80 ~ 89	134	80	< 0.001
> 90	14	10	< 0.001
Smoking	129 (30.0%)	401 (26.0%)	0.099
Hyperlipidemia	105 (24.4%)	296 (19.2%)	0.018
Diabetes mellitus	190 (44.0%)	201 (13.0%)	< 0.001
Hypertension	301 (70.0%)	485 (31.4%)	< 0.001
Coronary heart disease	203 (47.1%)		
LVEF	36.5 ± 14.9	70.3 ± 7.0	< 0.001

There are 431 HF-diagnosed patients in total with complete clinical data, and 1545 non-HF patients

Data presented as mean ± standard deviation or n (%)

*LVEF* left ventricular ejection fraction

p-value: T-test was applied for continuous data, while chi-square for categorical one

As depicted in Supplementary Fig. 1, the first dataset was obtained from the research [20]. The primary objective of this study was to examine hemodynamic indicators in individuals who have been diagnosed with heart failure. In the current investigation on heart failure, a cohort of 80 patients who satisfied the criteria specified in the Heart Failure guidelines were initially included. According to the most recent findings derived from our ongoing research, the dataset has been expanded to include over 400 instances. To effectively achieve the aims of our research, we acquired pressure waveforms exclusively from a cohort comprising 431 patients who were diagnosed with heart failure based on the established Heart Failure criteria.

The second dataset was obtained from a previous research [26]. The initial sample size for the “Cardiovascular Disease Risk Factors Two-Township Study” (CVDFACTS) consisted of 2211 participants. A total of 1545 individuals without cardiovascular disease were identified as the ‘non-HF’ group in accordance with the provided data.

Among the 431 HF cases, most had undergone three to four rounds of follow-up (maximum: 13 rounds), and the PWs from the same patient were marked from V1 to V13 according to time order. At the meantime, 158 cases were removed from 1545 control cases due to loss of PW record. Consequently, 1521 and 1387 PWs were recorded from among the 431 patients with HF and 1545 control participants, respectively (Supplementary Fig. 1A). We collected 2908 PWs in total and clinical data, including age, sex, and other variables, from patients who volunteered to participate in clinical observational studies conducted at Taipei Veteran Hospital.

Participants all received transthoracic echocardiography performed by an experienced sonographer. All images were acquired using a commercially available machine (HD11 XE Ultrasound system, Koninklijke Philips N.V.) and digitized using the TomTec Image-Arena™ Software 4.0 (TomTec Imaging Systems GmbH, Munich, Germany) by the same sonographer. Left ventricular (LV) volume was acquired by tracing the endocardial border of the left ventricle at both the end-diastole and end-systole, then summing up a stack of elliptical disks in apical 4-chamber view. The determination of LV ejection fraction (EF) involved calculating the discrepancy between the volume of the left ventricle at the end of diastole and the volume at the end of systole.

### Carotid PWs preprocessing

The raw PWs obtained by applanation tonometry were continuous waveforms with a sampling rate of 500 Hz. Each raw continuous PW was ensemble-averaged and resampled by interpolation into 100 pressure points (Supplementary Fig. 2), resulting in one PW with the extracted features. Each PW was then normalized with a trough and peak of 0 and 1, respectively. Considering that our goal was to distinguish patients with HF from those without HF according to PW features, PWs from the same patients in different rounds were considered different cases in our DNN model. To avoid potential overfitting owing to the repeated PWs in different rounds derived from the same person, we kept the same person in either the training dataset or the testing dataset. Moreover, we performed a sensitivity analysis that included only baseline PWs from all subjects to construct the HF score.

### HF score: HF identification by DNN model

In this study, we constructed a one-dimensional convolution neural network model (Conv1D) to identify PWs from heart failure patients. The Conv1D structure is based on the structure from previous study and applied from Keras of TensorFlow [27, 28]. Our Conv1D model takes the PWs, which are one-dimensional records, as the input and outputs the HF score, which is the probability of the PW from a HF subject. The Conv1D architecture is depicted in Fig. 1A. Briefly, the model was first split into 12 branches with different kernel sizes and filters to extract features from PWs and concatenate the outputs from these 12 branches to generate the final predicted score: HF probability. The details of layers are described in Supplementary Fig. 3. Hyperparameters were set as default for maxpooling layer, with momentum as 0.9, epsilon as 1e-5 and axis as 1 for batch-normalization and those for model compiling like optimizer was set as ‘adam’, with the loss set as ‘binary_crossentropy' and the metrics set as ‘accuracy’. Additionally, we constructed an alternative neural network model with only hidden layer, or fully-connected layer (FCL, Fig. 1B) [29], to approve the effectiveness of the Conv1D model.

We initially applied 10 iterations of the hold-out cross-validation, a method employed to address the risk of sample bias resulting from overfitting by dividing the data into portions for training and testing. Each hold-out process randomly selected 80% of data to train the model and the remaining 20% of data to evaluate the constructed model on unseen data, noted as the train-to-test ratio of 8:2.

### Shapley Additive exPlanations: feature importance analysis

It is actually the same concept shared by DNN and traditional machine learning, like SVM (support vector machine) and BN (Bayesian network), to establish the dependencies between the inputs and the outputs; but it is much harder to explain those of the DNN, due to uncertainties calculated in black-box. We employed the Shapley Additive exPlanations (SHAP) algorithm to analyze the constructed DNN model and locate the important sections on the PW that affect the prediction. SHAP is an agnostic technique that analyzes feature importance [30, 31], and aligns with the underlying objective of the Shapley method, which aims to comprehend the impact of each individual factor on the final outcome.

Using all 2908 PWs as training data to build a final model classifying patients into those with versus without HF, we employed SHAP to locate the important PW sections that affected the prediction results. Because of the high time and space complexity of implementing SHAP with 2908 PWs, 100 PWs—57 HF patients and 43 non-HF participants—were randomly chosen as the background dataset. Another 100 PWs—44 from HF patients and 56 from non-HF participants—were used as the sample data. SHAP analyzed the impacts of 100 features on the HF score, which the model predicted for the sample PWs by replacing each with the value in the background dataset. We set the *nsample* (parameter in SHAP) to 2000, which made the SHAP resample each feature 2000 times to observe the oscillation of the probability score, and the impact of each feature was evaluated as the SHAP value, a SHAP measurement index.

### Cox model: integrative survival analysis

The Cox proportional hazard model [32], known as multivariate semi-parametric survival analysis, is a powerful statistical tool for testing whether several factors are independently related to the rate of a specific event [33, 34]. A preliminary step is to assess the assumption of risk proportionality, which assumes that the hazard ratio (HR) associated with the risk factor must be constant over time [35]. To demonstrate the effect of PWs on HR, we used the HF score derived from PWs and the other 83 clinical variables to define the Cox regression. The concordance index (C-index), which was popularized by Harrell [36], was used as a measure of goodness-of-fit for the Cox model, as was the Akaike information criterion (AIC) and log-likelihood ratio tests. The Cox regression model, called CoxPHFitter, was imported from the package “lifelines” in Python, and the “penalizer” of the parameter was set to 0, meaning no penalty on correlations between covariates during the fitting.

In the 2nd study for prognostic utility, HF scores derived by the DNN model from 387 HF patients in stable clinical condition along with other intact 83 clinical parameters were considered (Supplementary Fig. 1B). To choose the model inputs used in the Cox model, we first conducted a univariate Cox regression analysis of all candidate variables (Supplementary Table 1) to predict the risk of HF mortality along with rehospitalization. A total of 111 events occurred among the patients with HF. We excluded extremely imbalanced categorical variables, such as medication history of non-vitamin K antagonist oral anticoagulants, which had barely any positive cases. As indicated in Supplementary Table 2, we identified 15 significant parameters through univariate Cox regression analysis in addition to the HF score. These parameters were then included in the subsequent multivariable Cox regression analysis using a stepwise selection approach. We also performed a sensitivity analysis using a DNN model trained with only 387 PWs at the baseline visit to evaluate the robustness of the results (Supplementary Fig. 4).

### Conventional HF risk prediction models

To demonstrate the prognostic utility of the HF score, we used risk factors of other HF risk stratification models as parameters to run the Cox regression for comparisons: the Acute Decompensated Heart Failure National Registry (ADHERE) algorithm [37], Get With The Guidelines–Heart Failure (GWTG-HF) risk score [38], and Meta-Analysis Global Group in Chronic Heart Failure (MAGGIC) score [39]. In particular, ADHERE and GWTF-HF were originally designed to predict in-hospital mortality among inpatients. The performance of these three models was validated in previous cohort studies [40–44].

## Results

### Comparison between two groups

Table 1 reveals a notable disparity in gender distribution between our HF cases, with a higher proportion of men than women. In contrast, the gender distribution among non-HF subjects is more evenly balanced. On the other hand, HF cases are significantly older than those without HF, but the ratios under the age 50 and age 70 ~ 79 between the two group are somehow close (not reaching significance). The prevalence of hyperlipidemia, diabetes mellitus and hypertension are significantly higher in HF cases, implying their correlations with the occurrence of heart failure. LVEF (left ventricular ejection fraction) is significantly lower in HF cases, which is consistent with the assumption that LVEF reflects the function of heart [49].

### DNN model to identify patients with HF

**Fig. 2:**
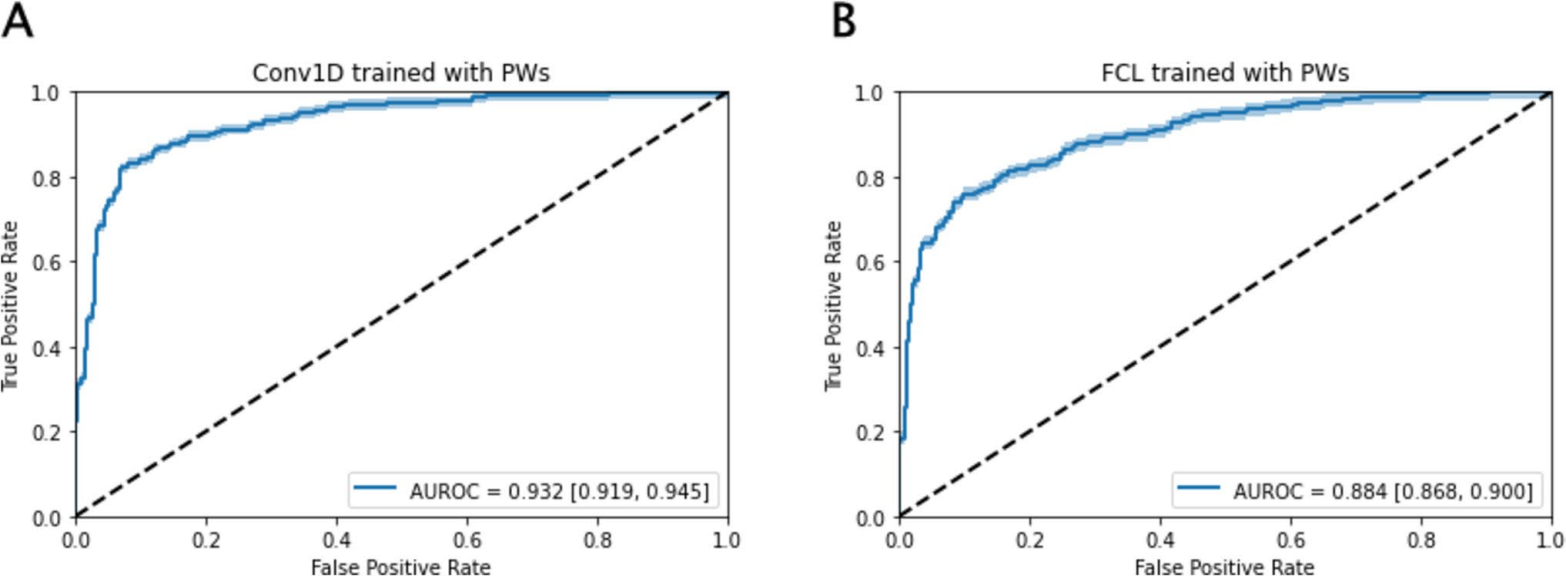
ROC of classification between HF and non-HF patients in hold-out cross-validation, with 95% CI in shadow. **A** Prediction by Conv1D trained with PWs (0.932, [0.919, 0.945]). **B** FCL trained with PWs (0.884, [0.868, 0.900])

As shown in Table 2, using 2908 PWs as input to train the Conv1D model under a train-to-test ratio of 8:2 in hold-out cross-validation with ten iterations, the average area under receiver operating characteristic curve (AUROC) is 0.932 (95% confidence interval [CI], 0.919–0.945; Fig. 2A). We also applied FCL to train the PWs, and the AUROC is 0.884 (95% CI, 0.868–0.900; Fig. 2B). These outcomes show that the Conv1D model could recognize pivotal patterns in PWs between patients with and without HF, and while the FCL made good predictions as well, it was inferior to the intact Conv1D model.

**Table 2:** Performance comparison between 4 models

	Sensitivity	Specificity	Accuracy	AUROC	F1-score	MCC
DNN	0.867	0.851	0.874	0.935	0.878	0.722
LR	0.796	0.844	0.819	0.890	0.820	0.641
SVM	0.815	0.843	0.828	0.901	0.831	0.659
RF	0.780	0.792	0.785	0.871	0.792	0.572

The values are averages of 10 iterations under train-to-test ratio of 8:2

*DNN* Deep neural network, *LR* Logistic regression, *SVM* Support vector machine, *RF* Random forest, *MCC* Matthews correlation coefficient

As for the sensitivity analysis including only PWs at the baseline visit (Supplementary Fig. 4), a consistent classification capability of the DNN model in recognizing HF PWs is noted.

### Comparison of different models for identifying patients with HF

We also used conventional machine learning, including logistic regression (LR) [45], support vector machine (SVM) [46], and random forest (RF) [47], in 10 iterations of 8:2 hold-out cross-validation for comparison (Supplementary Fig. 5). The AUROC of LR and SVM were approximately 0.91, exhibiting comparable classification accuracy to our DNN model, whereas RF had an AUROC of approximately 0.88. Table 2 shows the model performance of these four models, showing that the DNN model outperformed the other machine learning models. Within Table 2, MCC represents the Matthews correlation coefficient. This coefficient takes into account all four parameters in the equation simultaneously, resulting in a comprehensive evaluation of a model.

$$MCC= \frac{TN \times TP - FN \times FP}{\sqrt{\left(TP+FP\right)\left(TP+FN\right)\left(TN+FP\right)\left(TN+FN\right)}}$$

**Fig. 3:**
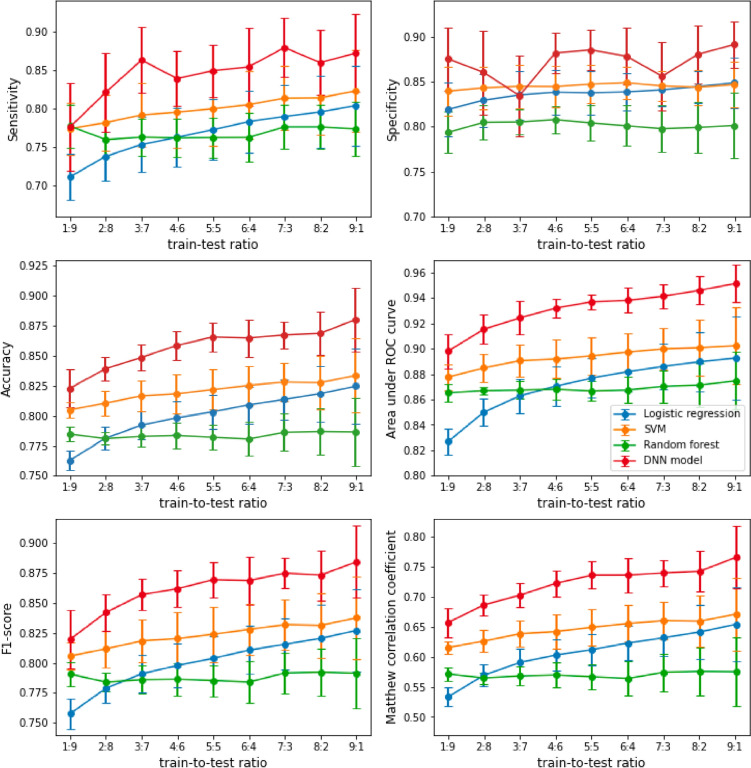
Comparative analysis of predictions using models and ratios, with plots generated using the mean values and 95% confidence intervals obtained from 10 iterations. We utilized six statistical methods, namely sensitivity, specificity, accuracy, AUROC, F1-score, and MCC, to evaluate the accuracy of the prediction models. Additionally, we tested the impact of the training dataset size by varying the train-to-test ratios from 1:9 to 9:1. In each plot, the y-axis represents the value, while the x-axis represents the varying ratio between training data and testing data. The four colors, namely blue, yellow, green, and red, represent logistic regression, support vector machines (SVM), random forest, and our deep neural network (DNN) model, respectively

A total of 2908 PWs were used as training data to construct the diagnostic models. We performed hold-out processes for PWs with 10 iterations over each train-to-test ratio, from 1:9 to 9:1, to demonstrate the robustness of the predictive power of the DNN model and compare the DNN and machine learning models (Fig. 3). According to the sensitivity, most of the models recognized patients with HF more accurately as the ratio increased, especially the DNN model (red) and LR model (blue). Meanwhile, the specificity of the DNN model remained approximately 0.85, with unstable fluctuations, whereas the machine learning model maintained steady levels. On the other hand, accuracy, AUROC, Matthew correlation coefficient (MCC), and F1-score show similar situations and demonstrate that the DNN model performs the best, reaching significance almost all the time, while SVM (orange) comes second.

These results demonstrate that our DNN model can identify PW patterns in patients with HF with sufficient input data and achieves even higher prediction accuracy with a larger dataset [48]; nevertheless, unstable specificity implies that a larger dataset does not solve the misrecognition problem of PW patterns in non-HF participants.

### Feature analysis to recognize pivotal characteristics of PWs

**Fig. 4:**
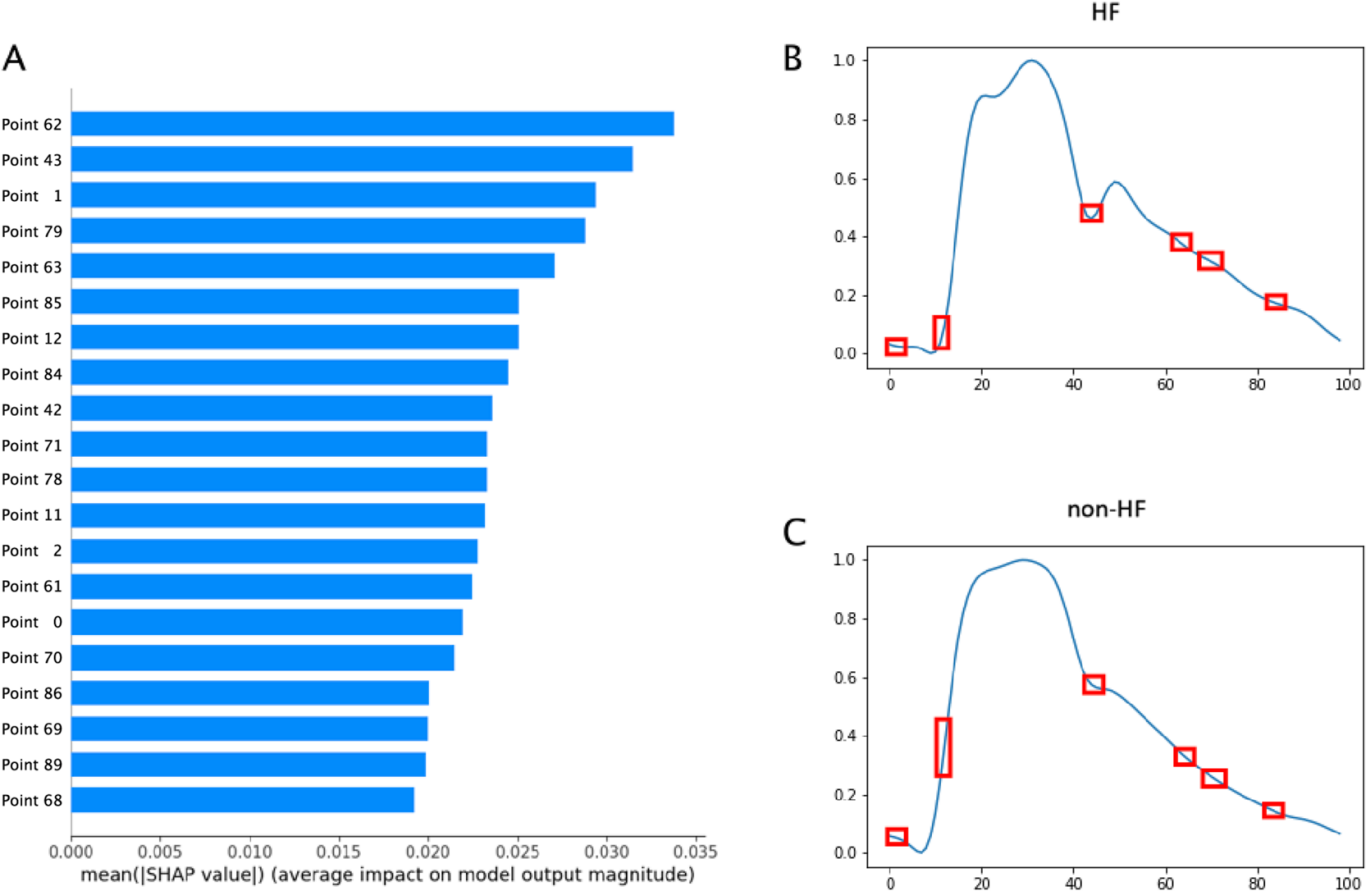
Outcomes of SHAP analysis. **A** Top 20 features with highest SHAP value; each feature corresponds to the relative location (100 points) on PW. One of **B** HF PWs and **C** non-HF PWs from 100 sample data; 6 sections with high SHAP values are framed in red for diagnosing HF due to their strong features

We used SHAP for the feature analysis using 100 randomly chosen PWs (HF:non-HF ratio of 57:43) and another 100 PWs (HF:non-HF ratio of 44:56) as the background dataset and sample data, respectively, without overlap. Feature values of the background dataset were substituted for those of the sample data 2000 times, and we obtained the top 20 of the 100 features with the highest SHAP value (Fig. 4A) along with the sum of the SHAP values of each feature (Supplementary Figs. 7E, F). According to the rank of SHAP values, seven portions with high SHAP values, such as the pre-systolic portion, which consisted of features from 0 to 2; the ascending systolic portion, from 11 to 12; the dicrotic notch part, from 42 to 43; and the descending diastolic portion, from 61 to 63, 68 to 71, and 84 to 86, exhibited important contributions to the DNN model (Figs. 4B, 4C).

The beta coefficients from LR and SVM were compared with the SHAP value (Supplementary Fig. 7), and some shared common important portions. The comparison of the top 20 SHAP values and beta coefficients (Supplementary Table 3) indicated that the pre-systolic (from 0 to 2) and end-diastolic (from 84 to 86) portions were common important features. Spearman correlation coefficients between these portions are shown in Supplementary Table 4.

### Application of HF score in risk stratification of HF patients

**Fig. 5:**
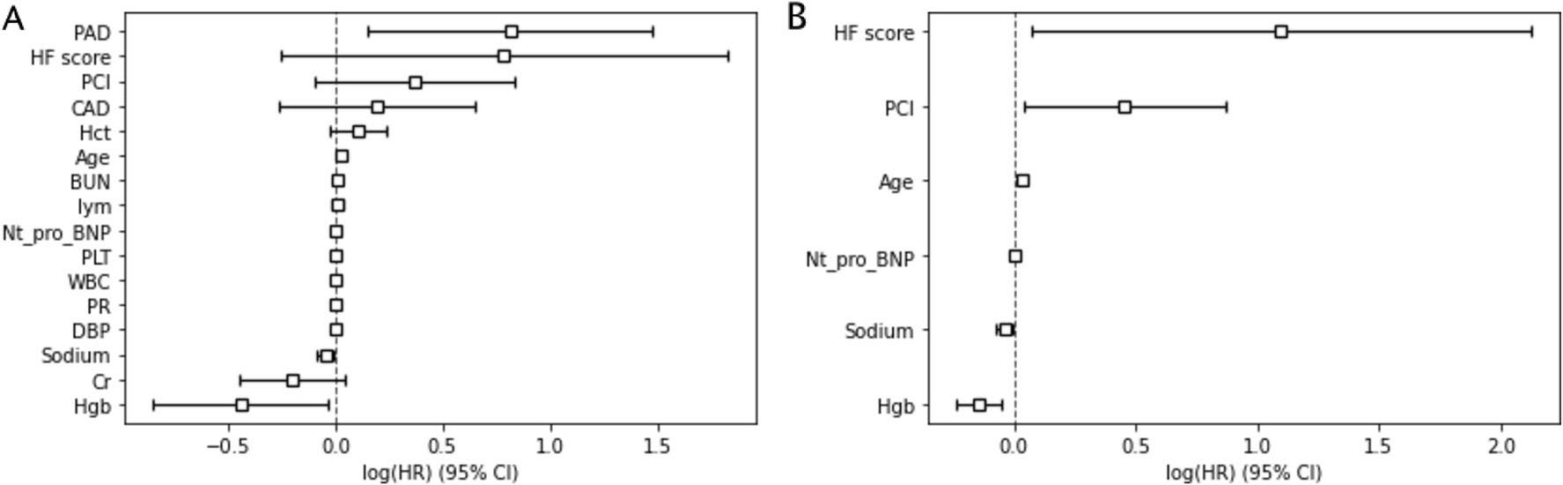
Results of Cox regression. **A** 16 variables and **B** 6 significant variables in multivariate analysis with logarithmic HR and 95% CI. *PAD* history of peripheral arterial disease, *PCI* history of percutaneous coronary intervention, *CAD* history of coronary arterial disease, *Hct* hematocrit, *BUN* blood urea nitrogen level, *Lym* lymphocyte level, *BUN* blood urea nitrogen level, *PR* pulse rate, *NT_pro_BNP* N-terminal pro-brain natriuretic peptide level, *PLT* platelet level, *WBC* white blood count, *DBP* diastolic blood pressure, *sodium* sodium level, *Hgb* hemoglobin level, *Cr* creatinine level

Using univariate Cox regression analysis, we obtained 15 significant variables and HF score (Supplemental Table 2). These 16 variables were then applied to a multivariate Cox regression model (Fig. 5A). Those no longer remaining significant in the multivariate model were excluded; only six variables were retained in the final model, the HF score model (Fig. 5B, Table 3), including the HF score, percutaneous coronary intervention (PCI), age, *N*-terminal pro-brain natriuretic peptide (NT-pro-BNP) concentration, sodium level in the emergency room, and hemoglobin (Hgb), by the order of hazard ratios. Based on the HF score, different predicted survival rates over time according to the different levels of the six parameters were plotted with reference to the baseline curve (Supplementary Fig. 6).

**Table 3:** A6 significant variables in multivariate analysis

Covariate	Hazard ratio [95% CI]	z	p	−log2(p)
﻿PCI	1.57 [1.04–2.38]	2.15	0.03	4.99
Hgb	0.87 [0.79–0.95]	−3.04	< 0.005	8.7
NT-pro-BNP	1.000013 [1.000002–1.000025]	2.39	0.02	5.87
sodium	0.96 [0.93–1.00]	−2.08	0.04	4.73
HF score	2.99 [1.07–8.35]	2.09	0.04	4.78
Age	1.03 [1.02–1.05]	4.21	< 0.005	15.24

Z-scores, p-values of them are shown

*PCI* history of percutaneous intervention, *Hgb* serum concentration of hemoglobin, *Nt-pro-BNP* N-terminal pro-brain natriuretic peptide level, *sodium* sodium level

**Fig. 6:**
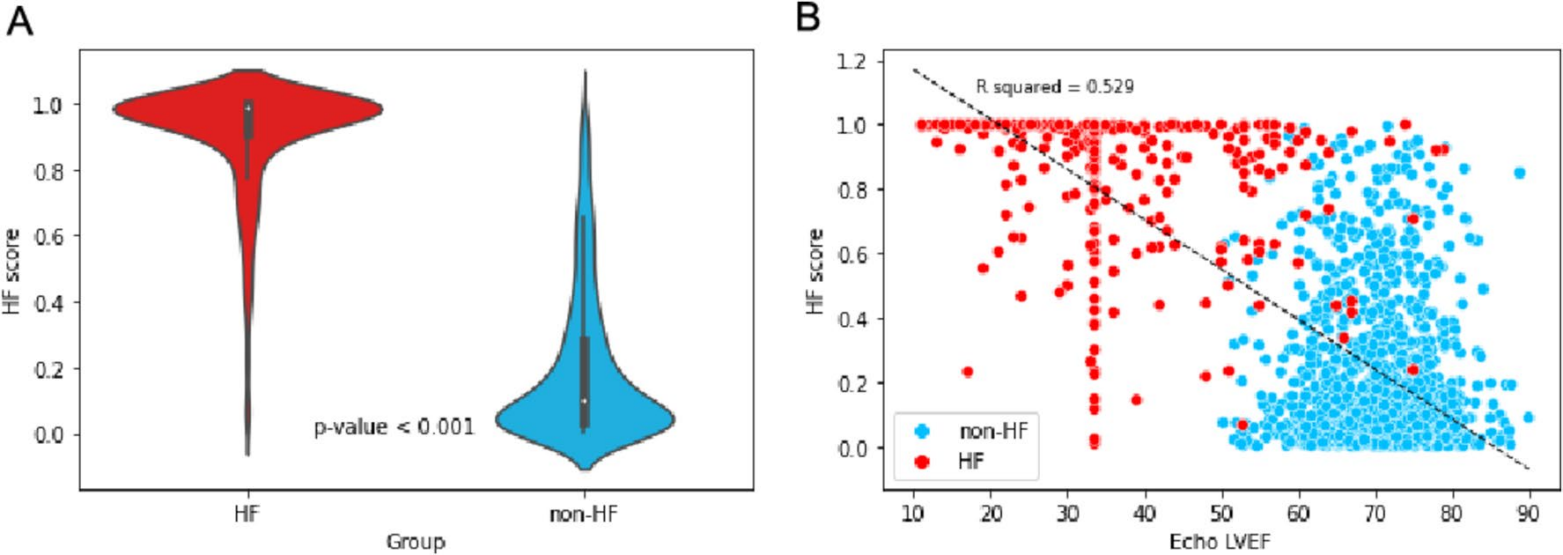
HF score analysis. **A** Comparison between groups of HF and non-HF cases in violin plot, p-value < 0.001. **B** Linear regression between HF score and Echo LVEF, with R^2^ = 0.529

We made a comparison between the HF scores for people with and without HF. The HF scores of individuals diagnosed with HF were found to be 0.910 ± 0.170, which exhibited a statistically significant increase compared to those individuals without HF (0.196 ± 0.227; p < 0.001). Furthermore, we conducted an evaluation of the relationship between the HF score and HF severity by examining the correlation between the HF score and the LV EF measured through echocardiography. The correlation analysis revealed a negative correlation coefficient of −0.233 (p < 0.001), indicating that the HF score does indeed vary in accordance with the diagnosis of HF and the severity of the condition. (Fig. 6B).

The final multivariable Cox model, known as the HF score model, demonstrated satisfactory performance by incorporating the HF score and five other factors. The factors were selected using a backward stepwise selection process from a pool of 15 parameters. All six factors in the model showed statistical significance, as indicated by the results in Table 3. In order to conduct a comparative analysis, we developed an alternative Cox model that excluded the HF score variable while keeping the other covariates intact. Incorporating the HF score into the predictive model resulted in enhanced performance, as evidenced by a higher log-likelihood ratio (59.11 compared to 54.06), a greater C-index (0.71 compared to 0.70), and a lower AIC value (1202.02 compared to 1205.07) compared to the model without the HF score. Besides, as shown in Table 4, the Cox model constructed using the parameters of the ADHERE algorithm had a C-index of 0.63. The C-indices based on parameters of two other risk prediction models were 0.68 and 0.69 for GWTG-HF and MAGGIC, respectively.

**Table 4:** Comparisons of model performance between different risk stratification models

Models	Parameters	C-indice
ADHERE	SBP, Cr, BUN	0.63
GWTG-HF	COPD, Age, BUN, SBP, PR, Sodium	0.68
MAGGIC	DM, Smoking, COPD, LVEF, ACEi, β-blocker, SBP, Cr, BMI, Age, BUN	0.69
HF score model	PCI, Hgb, Nt-pro-BNP, Sodium, Age, HF score	0.71

*ADHERE* Acute Decompensated Heart Failure National Registry, *GWTG-HF* Get With The Guidelines–Heart Failure, *MAGGIC* Meta-Analysis Global Group in Chronic Heart Failure, *SBP* Systolic blood pressure, *COPD* Chronic obstructive pulmonary disease, *DM* Diabetes mellitus, *ACEi* Angiotensin converting enzyme inhibitor, *BMI* Body mass index

## Discussion

Our study demonstrated that examining arterial pulsation and carotid PWs alone can identify and help with the risk stratification of patients with HF in clinical practice. The DNN model had an excellent classification ability to distinguish between HF and non-HF PWs after training with PW data. Conventional machine learning exhibited comparable abilities, especially SVM, even matching the DNN model in some situations. However, with an increasing amount of training data, the performance of the DNN model improved substantially, whereas that of the SVM stagnated at a certain level. These findings are in line with the concept that deep learning performs even better with larger datasets versus machine learning [48]. If a larger dataset can be collected to enhance the DNN model, the deep learning model is expected to be more helpful than machine learning in clinical practice.

The conventional noninvasive assessment of pulse waves, such as carotid-femoral pulse wave velocity and augmentation index, has been suggested as a useful clinical assessment of arterial aging and predictor of cardiovascular events from the past [50, 51] to the present [52, 53]. Nevertheless, aside from some investigations discussing the prognostic value of pulse pressure [54] and pulse wave velocity [55], advanced evaluations of the association between PWA and prognosis in patients with HF are scarce. As such, our study represents pioneering efforts as a novel approach obtained from the information provided by PWs to help improve the clinical care of patients with HF.

Regarding the important contributing features of PW to the HF score, the machine learning findings agreed with those obtained from our DNN model in some portions of the PW; nevertheless, the top 20 features between them seem quite different (Supplementary Table 3), and the Spearman correlation indicated that SHAP values were significantly correlated with neither the beta coefficients of LR nor those of SVM. This implies that machine learning and deep learning analyze the characteristics of PW in divergent ways, which may be the reason behind the DNN model surpassing machine learning under certain conditions. On the other hand, SHAP values suggested that the systolic portion of the PW had high negative importance (non-HF) and that the dicrotic notch along with the diastolic portion affected the positive prediction (HF) (Supplementary Fig. 7E). Given the unrevealed information about PW, the DNN model should have detected some important waveform features from the interactions between the upslope and downslope of PW. Therefore, future mechanistic studies are needed to reveal the relationships of these identified portions with HF and provide deeper insight [56–58].

In the Cox regression analysis, despite the fact HF score did not reach significant in univariate regression, the HF score was a significant predictor in the multivariate analysis (Table 3), which supports the valuable role of PWs in the management of HF patients. After removal of the HF score from the Cox regression, the C-index decreased and the AIC increased, supporting our conclusion that the HF score helps improve the prognostic accuracy in patients with HF. Additional comparison between HF scores of people with or without HF drew a conclusion that HF score does change with diagnosis of HF and the severity of it. Other clinical data, such as age, serum sodium level, NT-pro-BNP, Hgb, and history of PCI, were risk factors identified by the multivariate model. The identification of these established risk factors suggests the internal validity of our data.

Compared with other risk stratification models, we found that HF score model demonstrated a superior prognostic value and an even better than conventional risk prediction model according to the C-index, which was 0.71 for the HF score model and 0.63, 0.68, and 0.69 for the ADHERE, GWTG-HF, and MAGGIC, respectively. Regarding our six significant variables, sodium serum level is also a predictor in the GWTG-HF risk score, while age is commonly recognized as a predictor by the GWTG-HF and the MAGGIC. PCI is also a traditional risk factor for HF, and it suggests the comorbidity of coronary artery disease, which is associated with an increased mortality rate [59]. Finally, NT-pro-BNP is a remarkable risk predictor of mortality in patients with HF [60–62], which was confirmed here. Remarkably, blood urea nitrogen (BUN), serum creatinine (Cr), and pulse rate (PR) were statistically significant predictors in our univariate Cox regression and predictors in all three conventional models, but none were statistically significant in the multivariate model.

The novel findings of our study on the utility of PWs require external validation. However, our results will lead to new diagnostic opportunities in cardiovascular medicine by providing insight on the use of AI technology and its possible improvement. Further studies should be conducted to relate PW and PW-like waveforms, such as photoplethysmography, to more cardiovascular diseases and, in turn, uncover its corresponding pathophysiology.

### Limitations

PWs are limited to data originally collected from other cohorts [25], and future prospective cohort studies are required to validate our study findings. Due to the “black box problem” of deep learning [63], there is no certain way to explain the output from DNN model; this is also why used only SHAP to identify important PW portions. Applanation tonometry was used to obtain the PWs. Whether PWs acquired by other techniques, such as photoplethysmography or oscillometry, can result in comparable findings requires further confirmation.

## Conclusion

This study demonstrated the utility of carotid PW using a DNN model to identify possible cases of HF and assess the prognosis of these patients along with other conventional clinical data. These results suggest that arterial PWs contain important information about cardiovascular disease and can help improve HF management.

### Supplementary Information

Below is the link to the electronic supplementary material.

Supplementary file1 (DOCX 929 KB)

## Data Availability

The datasets during and/or analyzed during the current study available from the corresponding author on reasonable request.

## Abbreviations

PWA: Pulse wave analysis
HF: Heart failure
non-HF: Without heart failure
PP: Pulse pressure
PW: Pressure waveform
DNN: Deep neural network
CNN: Convolutional neural network
SVM: Support vector machine
BN: Bayesian network
CVDFACTS: Cardiovascular Disease Risk Factors Two-Township Study
Conv1D: 1-Dimension convolutional neural network
FCL: Fully-connected layer
HR: Hazard ratio
C-index: Concordance index
SHAP: Shapley Additive exPlanations
AUROC: Area under ROC curve
LR: Logistic regression
RF: Random forest
MCC: Matthew correlation coefficient
KM: Kaplan–Meier
AIC: Akaike information criterion
ADHERE: Acute Decompensated Heart Failure National Registry
GWTG-HF: Get With The Guidelines–Heart Failure
MAGGIC: Meta-Analysis Global Group in Chronic Heart Failure
PCI: Percutaneous Coronary Intervention
Nt-pro-BNP: *N*-Terminal pro-brain natriuretic peptide
Hgb: Hemoglobin
LVEF: Left ventricular ejection fraction
BUN: Blood urea nitrogen
Cr: Creatinine
PR: Pulse rate
SBP: Systolic blood pressure
COPD: Chronic obstructive pulmonary disease
DM: Diabetes mellitus
ACEi: Angiotensin converting enzyme inhibitor
BMI: Body mass index

## References

[1] Tripoliti EE (2017). Heart failure: diagnosis, severity estimation and prediction of adverse events through machine learning techniques. Comput Struct Biotechnol J.

[2] Gheorghiade M (2005). Acute heart failure syndromes—current state and framework for future research. Circulation.

[3] Roger VL (2021). Epidemiology of heart failure: a contemporary perspective. Circ Res.

[4] Bui AL, Horwich TB, Fonarow GC (2011). Epidemiology and risk profile of heart failure. Nat Rev Cardiol.

[5] Savarese G (2022). Global burden of heart failure: a comprehensive and updated review of epidemiology. Cardiovasc Res.

[6] Wilkinson IB, Cockcroft JR, Webb DJ (1998). Pulse wave analysis and arterial stiffness. J Cardiovasc Pharmacol.

[7] Wojciechowska WC (2002). Analiza fali tetna: od podstaw do zastosowań [Pulse wave analysis: from the basic sciences to clinical applications]. Przegl Lek.

[8] Nelson MR (2010). Noninvasive measurement of central vascular pressures with arterial tonometry: clinical revival of the pulse pressure waveform?. Mayo Clin Proc.

[9] Drzewiecki GM, Melbin J, Noordergraaf A (1983). Arterial tonometry—review and analysis. J Biomech.

[10] Chen CH (1996). Validation of carotid artery tonometry as a means of estimating augmentation index of ascending aortic pressure. Hypertension.

[11] Mitchell GF, Pfeffer MA (1999). Pulsatile hemodynamics in hypertension. Curr Opin Cardiol.

[12] DeLoach SS, Townsend RR (2008). Vascular stiffness: its measurement and significance for epidemiologic and outcome studies. Clin J Am Soc Nephrol.

[13] Kawasaki T (1987). Noninvasive assessment of the age-related-changes in stiffness of major branches of the human arteries. Cardiovasc Res.

[14] Benetos A (1993). Arterial alterations with aging and high blood-pressure—a noninvasive study of carotid and femoral arteries. Arterioscler Thromb.

[15] O'Rourke MF (2016). Carotid artery tonometry: pros and cons. Am J Hypertens.

[16] Weber T, Chirinos JA (2018). Pulsatile arterial haemodynamics in heart failure. Eur Heart J.

[17] Mitchell GF (2001). Pulsatile hemodynamics in congestive heart failure. Hypertension.

[18] Laskey WK, Kussmaul WG (1987). Arterial wave reflection in heart-failure. Circulation.

[19] Westerhof N, Orourke MF (1995). Hemodynamic basis for the development of left-ventricular failure in systolic hypertension and for its logical therapy. J Hypertens.

[20] Sung SH (2011). Pulsatile hemodynamics and clinical outcomes in acute heart failure. Am J Hypertens.

[21] Lee H, Song J (2019). Introduction to convolutional neural network using Keras; an understanding from a statistician. Commun Stat Appl Methods.

[22] Bohr AM, K.,  (2020). The rise of artificial intelligence in healthcare applications. Artificial intelligence in healthcare.

[23] Davenport TKR (2019). The potential for artificial intelligence. Future Healthc J.

[24] Kiranyaz S (2021). 1D convolutional neural networks and applications: a survey. Mech Syst Signal Process.

[25] Ali H (2019). Imbalance class problems in data mining: a review. Indones J Electr Eng Comput Sci.

[26] Cheng HM (2016). Prognostic significance of mechanical biomarkers derived from pulse wave analysis for predicting long-term cardiovascular mortality in two population-based cohorts. Int J Cardiol.

[27] Raghunath S (2021). Deep neural networks can predict new-onset atrial fibrillation from the 12-Lead ECG and help identify those at risk of atrial fibrillation-related stroke. Circulation.

[28] Hsieh CH (2020). Detection of atrial fibrillation using 1D convolutional neural network. Sensors.

[29] Touretzky DS, Pomerleau DA (1989). What’s hidden in the hidden layers. Byte.

[30] Kumar CSC (2020). Dimensionality reduction based on SHAP analysis: a simple and trustworthy approach. Int Conf Commun Signal Process.

[31] Parsa AB (2020). Toward safer highways, application of XGBoost and SHAP for real-time accident detection and feature analysis. Accid Anal Prev.

[32] Cox DR (1972). Regression models and life-tables. J Roy Stat Soc.

[33] Katz MH, Hauck WW (1993). Proportional hazards (Cox) regression. J Gen Intern Med.

[34] Kumar D, Klefsjo B (1994). Proportional hazards model—a review. Reliab Eng Syst Saf.

[35] Abd ElHafeez S (2021). Methods to analyze time-to-event data: the Cox regression analysis. Oxid Med Cell Longev.

[36] Harrell FE, Lee KL, Mark DB (1996). Multivariable prognostic models: issues in developing models, evaluating assumptions and adequacy, and measuring and reducing errors. Stat Med.

[37] Fonarow GC (2005). Risk stratification for in-hospital mortality in acutely decompensated heart failure—classification and regression tree analysis. JAMA.

[38] Peterson PN (2010). A validated risk score for in-hospital mortality in patients with heart failure from the American Heart Association get with the guidelines program. Circ Cardiovasc Qual Outcomes.

[39] Pocock SJ (2013). Predicting survival in heart failure: a risk score based on 39 372 patients from 30 studies. Eur Heart J.

[40] Win SH, Hebl I, Redfield V, M. M.  (2015). Abstract 12302: mortality and readmissions after heart failure hospitalization in a community based cohort: estimating risk using the acute decompensated heart failure national registry (ADHERE) classification and regression tree (CART) algorithm. Circulation.

[41] Sartipy U (2014). Predicting survival in heart failure: validation of the MAGGIC heart failure risk score in 51,043 patients from the Swedish heart failure registry. Eur J Heart Fail.

[42] Suzuki S (2018). Clinical significance of get with the guidelines-heart failure risk score in patients with chronic heart failure after hospitalization. J Am Heart Assoc.

[43] Lagu T (2016). Validation and comparison of seven mortality prediction models for hospitalized patients with acute decompensated heart failure. Circ Heart Fail.

[44] Passantino A (2015). Predicting mortality in patients with acute heart failure: role of risk scores. World J Cardiol.

[45] DeMaris A (1995). A tutorial in logistic regression. J Marriage Fam.

[46] Noble W (2006). What is a support vector machine?. Nat Biotechnol.

[47] Rigatti SJ (2017). Random forest. J Insur Med.

[48] Laqtib S, El Yassini K, Hasnaoui ML (2019) A deep learning methods for intrusion detection systems based machine learning in MANE*T.* In: 4th International conference on smart city applications (Sca' 19)

[49] Hajouli S, Ludhwani D (2023). Heart failure and ejection fraction.

[50] Laurent S (2006). Expert consensus document on arterial stiffness: methodological issues and clinical applications. Eur Heart J.

[51] Chae CU (1999). Increased pulse pressure and risk of heart failure in the elderly. JAMA.

[52] Townsend RR (2015). Recommendations for improving and standardizing vascular research on arterial stiffness: a scientific statement from the American Heart Association. Hypertension.

[53] Bruno RM (2020). Early and supernormal vascular aging clinical characteristics and association with incident cardiovascular events. Hypertension.

[54] Domanski MJ (1999). Independent prognostic information provided by sphygmomanometrically determined pulse pressure and mean arterial pressure in patients with left ventricular dysfunction. J Am Coll Cardiol.

[55] Regnault V (2014). Opposite predictive value of pulse pressure and aortic pulse wave velocity on heart failure with reduced left ventricular ejection fraction insights from an eplerenone post-acute myocardial infarction heart failure efficacy and survival study (EPHESUS) substudy. Hypertension.

[56] Wilkinson IB (2000). The influence of heart rate on augmentation index and central arterial pressure in humans. J Physiol-Lond.

[57] Tartiere JM (2006). Interaction between pulse wave velocity, augmentation index, pulse pressure and left ventricular function in chronic heart failure. J Hum Hypertens.

[58] Xia JL, S.  (2018). Pulse wave analysis for cardiovascular disease diagnosis. Digit Med.

[59] Lala A, Desai AS (2014). The role of coronary artery disease in heart failure. Heart Fail Clin.

[60] Hartmann F (2004). Prognostic impact of plasma N-terminal pro-brain natriuretic peptide in severe chronic congestive heart failure: a substudy of the carvedilol prospective randomized cumulative survival (COPERNICUS) trial. Circulation.

[61] Sawano M (2018). Performance of the MAGGIC heart failure risk score and its modification with the addition of discharge natriuretic peptides. ESC Heart Fail.

[62] Khanam SS (2018). Validation of the MAGGIC (meta-analysis global group in chronic heart failure) heart failure risk score and the effect of adding natriuretic peptide for predicting mortality after discharge in hospitalized patients with heart failure. PLoS ONE.

[63] Hussain J (2019) Deep learning black box problem. Master’s thesis, Uppsala University: Uppsala, Sweden, Department of Informatics and Media

