# Supplementary material for: Examining arterial pulsation to identify and risk-stratify heart failure subjects with deep neural network
Source: Phys Eng Sci Med. 2024 Feb 15;47(2):477–89. doi: 10.1007/s13246-023-01378-6 (PMC11166827; doi:10.1007/s13246-023-01378-6)
Supplement: Supplementary file 1 — Supplementary file1 (DOCX 929 KB) [file 13246_2023_1378_MOESM1_ESM.docx]

**Supplemental file**

**Supplementary Figure 1.** Flowcharts of data selection for analyses continued.

**A,** Total 2908 PWs for DNN model. **B,** Total 387 HF scores from HF patients with clinical data for Cox regression.

HF: Heart failure

CVDFACTS: Cardiovascular Disease Risk Factors Two-Township Study

**Supplementary Figure 2.** Resampling pressure waveform.

The original ensemble average PW of the subject HF001V2 was resampled into 100 points of features by interpolation, and preprocessed by min-max normalization for DNN model.


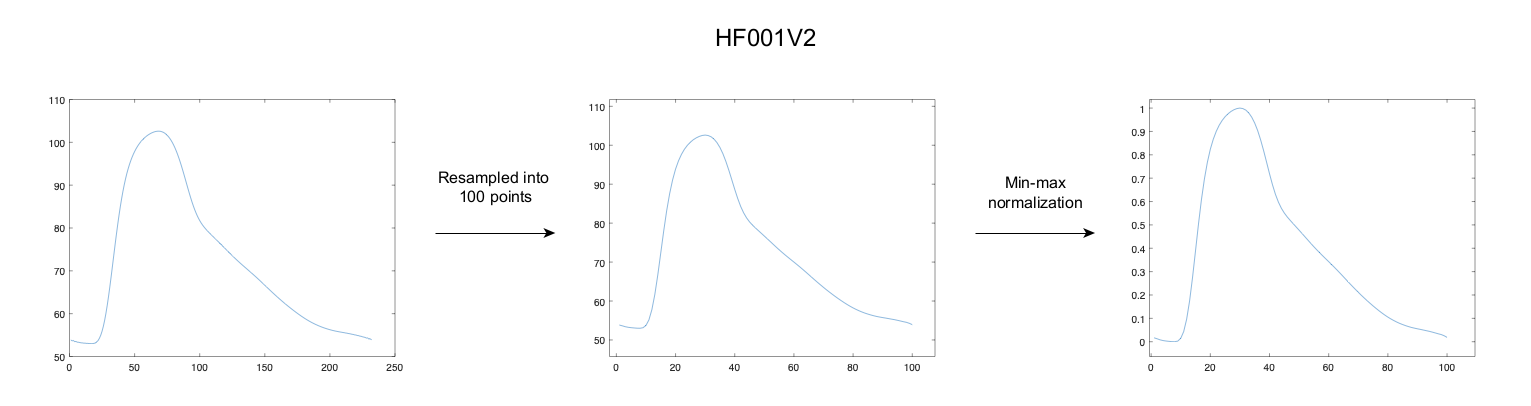


**Supplementary Figure 3**. Functions of layers in deep neural networks.

**A,** One-D-convolution extracts features from input data by individual weights on kernel and projects the outcomes into the output layer, whose size is set by filters; the example below sets the parameters as kernel_size = 3, filters = 7 (default strides = 1).

**B,** Maxpooling layer catches the biggest number as its behalf of each sliding window; the default parameters are pool_size = 2 and strides = 2.

**C,** Batch-normalization is a transformation, by the unit of batch_size (we set it as 32), that maintains the mean output close to 0 and the output standard deviation close to 1, which can be altered by γ and β (set as 1 and 0 in default).

**D,** Dense layer, or fully-connected layer (referred to as FCL in this study), receives outputs from preceding layers and produces new outputs through activation, which was set as “ReLU” in most of our DNN layers except for the last 1-unit dense layer as “Sigmoid”. The example below shows connections between 3 dense layers.

**E,** Flatten layer rearranges the tensor, the vector information in neural network, into the one-dimensional shape of its size.


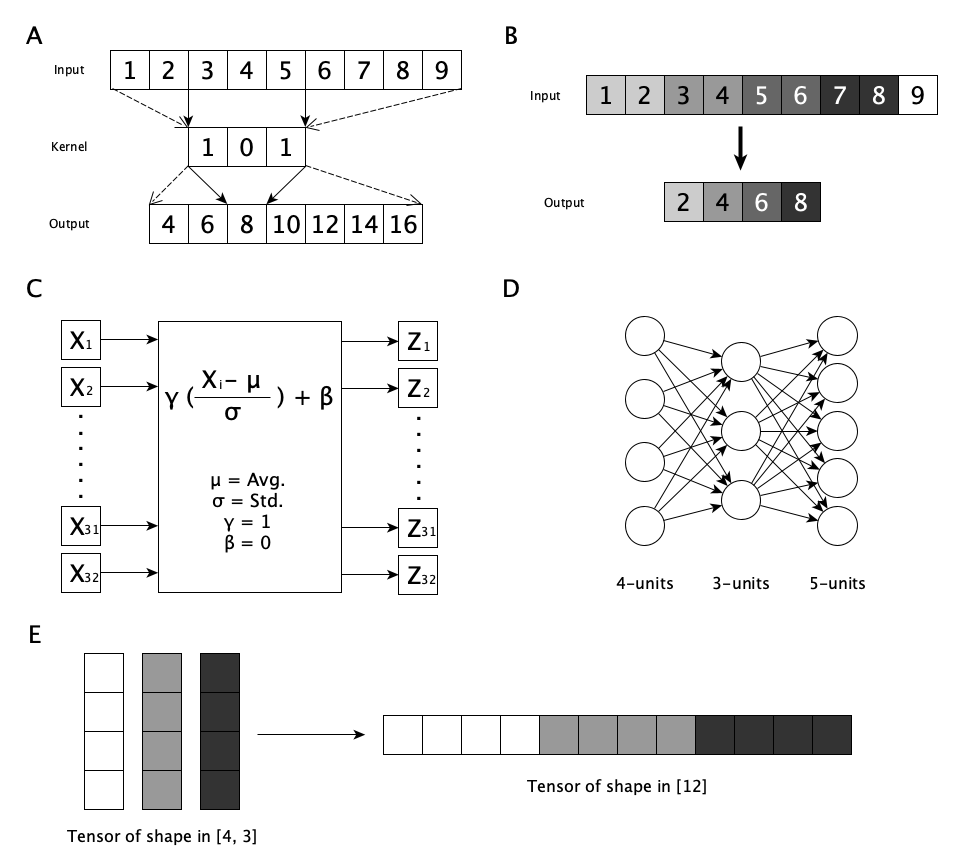


**Supplementary Figure 4.** Sensitivity test by DNN model at baseline visit.

A same structure of DNN model but trained with only 387 V2 HF and 1387 non-HF PWs. **A,** Prediction by Conv1D trained with PWs (0.944, [0.954, 0.934]). **B,** FCL trained on PWs (0.916, [0.906, 0.926]).

**Supplementary Figure 5.** ROC of each machine learning.

**A,** Logistic regression, 95% CI = [0.867, 0.913]. **B,** SVM (Support vector machine), 95% CI = [0.879, 0.923]. **C,** Random forest, 95% CI = [0.872, 0.889].

**Supplementary Figure 6.** Predicted survival rate over time.

Survival probability decreases with days in 6 variable values, calculated by Cox proportional hazard model; the baseline curve is dashed and plotted with each covariate as the median.


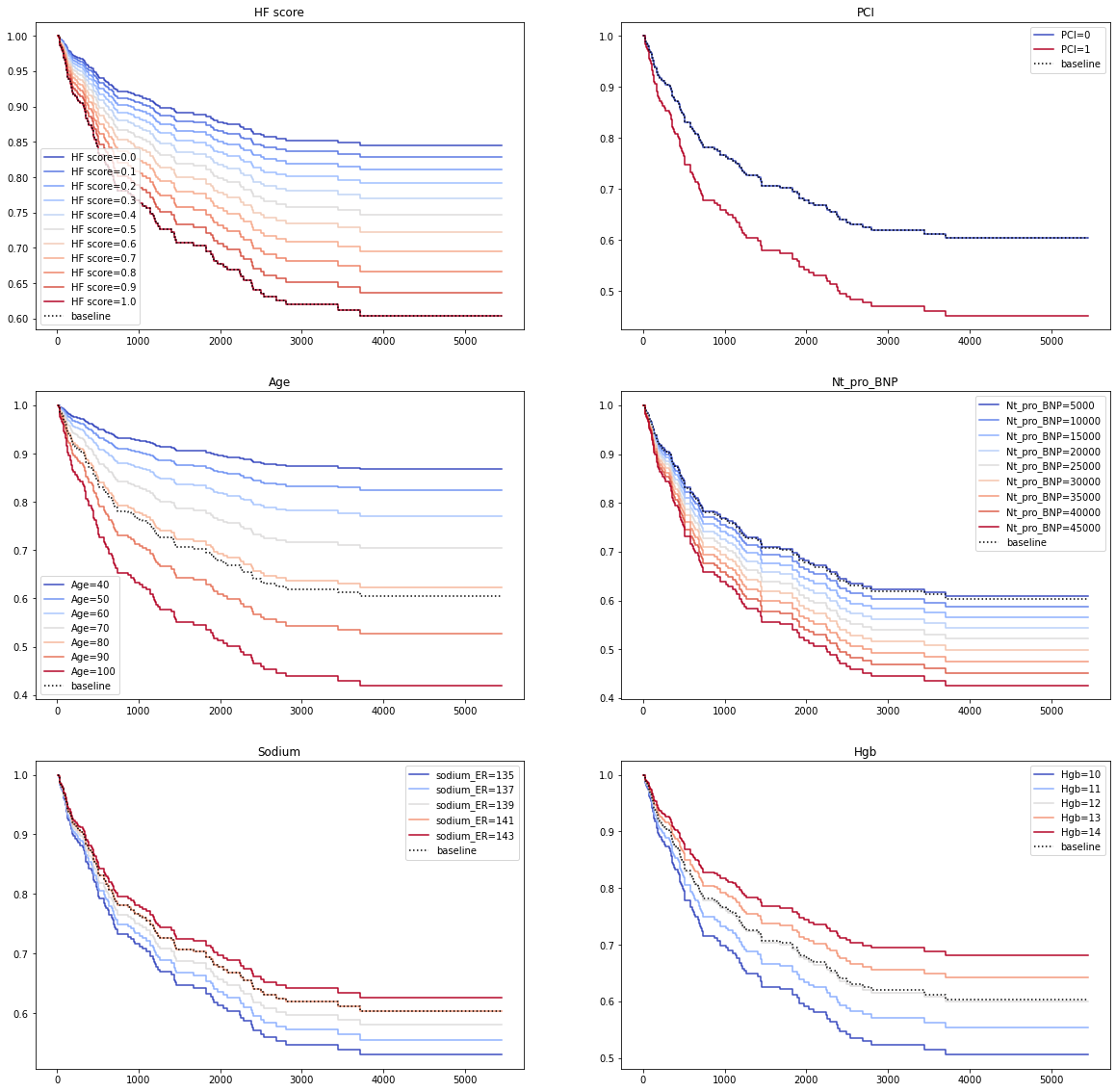


**Supplementary Figure 7**. Visualization of feature importance between models.

Beta-coefficients under machine learning models of **A,** LR with **B,** its absolute value and **C,** SVM with **D,** its absolute value; sum of **E,** SHAP values and **F,** absolute SHAP values of each feature from SHAP analysis of DNN model, in which F is corresponding to Figure 9A. The positive/negative value means effect on HF/non-HF prediction.

**Supplementary table 1.** 84 variables of 387 HF patients.

6 significant variables in multivariate Cox regression are highlighted in gray boxes.

**Supplementary table 2.** 16 statistically significant variables in univariate Cox regression.

6 variables (including HF score) retained for multivariate Cox regression are highlighted in gray boxes.

exp(coef) = hazard ratio

**Supplementary table 3.** Top 20 important features of models.

Comparison between top 20 absolute beta-coefficients of LR, SVM and SHAP values of DNN model, among 100 features in PW. Common top 20 features are highlighted with gray background.

| Rank | LR | SVM | DNN |
| --- | --- | --- | --- |
| 1 | 87 | 86 | 62 |
| 2 | 86 | 87 | 43 |
| 3 | 0 | 0 | 1 |
| 4 | 8 | 85 | 79 |
| 5 | 7 | 88 | 63 |
| 6 | 88 | 99 | 85 |
| 7 | 85 | 1 | 12 |
| 8 | 1 | 84 | 84 |
| 9 | 99 | 98 | 42 |
| 10 | 98 | 2 | 71 |
| 11 | 84 | 7 | 78 |
| 12 | 2 | 89 | 11 |
| 13 | 89 | 8 | 2 |
| 14 | 9 | 97 | 61 |
| 15 | 97 | 96 | 0 |
| 16 | 6 | 83 | 70 |
| 17 | 83 | 6 | 86 |
| 18 | 96 | 90 | 69 |
| 19 | 90 | 95 | 89 |
| 20 | 14 | 3 | 68 |

**Supplementary table 4.** Spearman correlations between models.

Spearman correlation between absolute SHAP values (Supplementary Figure 7F for DNN) and beta-coefficients (Supplementary Figure 7B for LR, Supplementary Figure 7D for SVM) among 100 PW features.

| Spearman | correlation | p-value |
| --- | --- | --- |
| LR & DNN | 0.067 | 0.5006 |
| SVM & DNN | 0.109 | 0.2746 |
| LR & SVM | 0.750 | < 0.001 |
